# Experimental evaluation of direct thromboaspiration efficacy according to the angle of interaction between the aspiration catheter and the clot

**DOI:** 10.1136/neurintsurg-2020-016889

**Published:** 2021-01-22

**Authors:** Gianmarco Bernava, Andrea Rosi, José Boto, Jeremy Hofmeister, Olivier Brina, Philippe Reymond, Michel Muster, Hasan Yilmaz, Zsolt Kulcsar, Emmanuel Carrera, Mohamed Bouri, Karl-Olof Lovblad, Paolo Machi

**Affiliations:** 1 Division of Neuroradiology, Geneva University Hospitals, Geneva, Switzerland; 2 Division of Neuroradiology, University Hospital Zurich, Zurich, Switzerland; 3 Division of Neurology, Geneva University Hospitals, Geneva, Switzerland; 4 Federal Institute of Technology of Lausanne (EPFL), Lausanne, Switzerland

**Keywords:** stroke, thrombectomy, device, stent

## Abstract

**Background:**

Successful direct thromboaspiration (DTA) is related to several factors such as clot consistency, size, and location. It has also been demonstrated recently that the angle of interaction (AOI) formed by the aspiration catheter and the clot is related to DTA efficacy. The aims of this study were three-fold: (a) to confirm the clinical finding that the AOI formed by the aspiration catheter and the clot influence DTA efficacy; (b) to evaluate to what extent this influence varies according to differences in clot consistency and size; and (c) to validate stent retriever thrombectomy as an effective rescue treatment after DTA failure in the presence of an unfavorable AOI.

**Methods:**

A rigid vascular phantom designed to reproduce a middle cerebral artery trifurcation anatomy with three M2 segments forming different angles with M1 and thrombus analog of different consistencies and sizes was used.

**Results:**

DTA was highly effective for AOIs >125.5°, irrespective of thrombus analog features. However, its efficacy decreased for acute AOIs. Rescue stent retriever thrombectomy was effective in 92.6% of cases of DTA failure.

**Conclusions:**

This in vitro study confirmed that the AOI formed by the aspiration catheter and the thrombus analog influenced DTA efficacy, with an AOI >125.5° related to an effective DTA. Stent retriever thrombectomy was an effective rescue treatment after DTA failure, even in the presence of an unfavorable AOI.

## Introduction

Direct thromboaspiration (DTA) is an effective technique for the endovascular treatment of acute ischemic stroke. However, in certain cases, DTA fails in clot removal and a stent retriever (STR) thrombectomy has to be performed as a rescue technique.^1–3^ Different factors are related to DTA efficacy, such as clot consistency, size, and location.^4–8^ A recent clinical study also observed that the angle of interaction (AOI) formed by the aspiration catheter (AC) and the clot was related to DTA efficacy, with values ≤125.5° associated with DTA failure. In addition, the study showed that STR thrombectomy was an effective rescue treatment in cases of DTA failure due to unfavorable AOIs.^9^

The aims of this in vitro study were three-fold: (a) to confirm the clinical finding that the AOI formed by the AC and the clot influenced DTA efficacy; (b) to evaluate to what extent the influence of the AOI varied according to differences in other factors such as clot consistency and size; and (c) to validate STR thrombectomy as an effective rescue treatment after DTA failure in the presence of an unfavorable AOI.

## Methods

Experiments were conducted using a rigid vascular phantom designed to reproduce a middle cerebral artery trifurcation anatomy and thrombus analog (TA) of different consistencies and sizes.

### Vascular phantom

**Figure 1:**
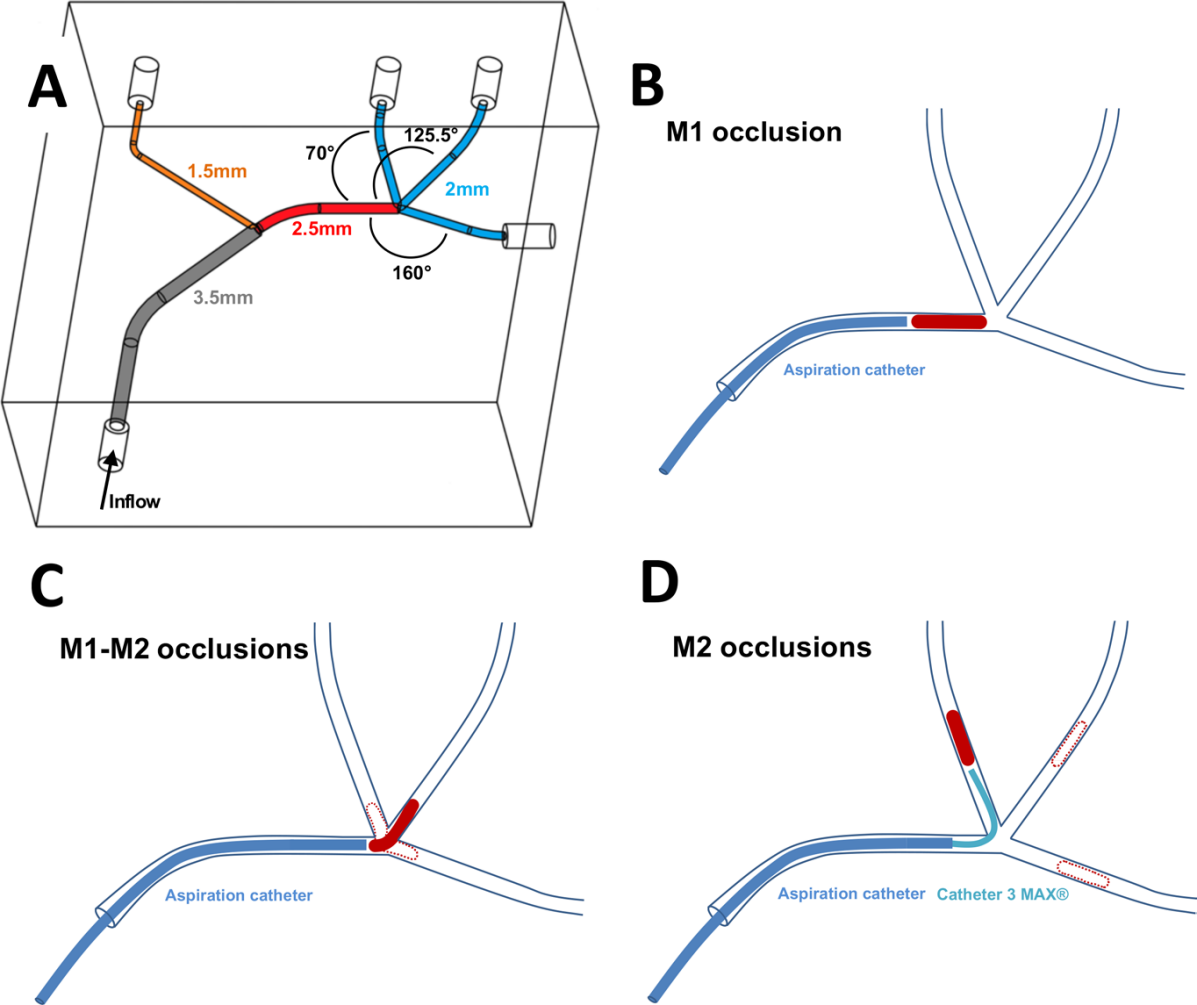
(A) Schematic representation of the vascular phantom used for the experiments, with vessel diameters and angles between M1 and M2 branches. (B) Thrombus analog location and catheter positioning for M1 occlusion experiments. (C) Location of thrombus analogs and catheter positioning for M1-M2 occlusion experiments. (D) Location of thrombus analogs and catheter positioning for M2 occlusion experiments.

The vascular phantom was carved from a block of polymethylmethacrylate using a computed numerical control machine to reproduce a middle cerebral artery with one M1 segment and three M2 segments. M2 segments formed an angle of 70°, 160°, and 125.5° with M1, respectively (figure 1A). The aim was to reproduce different AOIs between the AC placed in M1 and the TA placed in the respective M2 segment. The angle M1-M2/125.5° was identified in the clinical study as a threshold for DTA efficacy with angles ≤125.5° prone to DTA failure. The angles M1-M2/70° and M1-M2/160° were conceived for the present study in order to reproduce unfavorable and favorable AOIs, respectively. The choice of a rigid vascular phantom was made in order to prevent a modification of the anatomy potentially encountered by using a soft model.

### TA production

TAs were produced using mixtures of guar, borax, and water in order to mimic human clots of different consistencies. Guar and borax were added to water in different concentrations in order to obtain soft and hard mixtures. Specifically, 1.5 or 2 g of each element were mixed with 100 mL water to produce soft or hard TAs, respectively. The aim of such experimental TA production was to obtain reproducible consistencies through several series of experiments. Production samples of cylindrical shape with a diameter of 10 mm and a height of 3 mm were then extracted from the mixtures to verify their consistency by compression tests. Samples were compressed up to 50% of their initial height. Mixtures demanding compression forces ranging between 5.7 and 4.4 mN/mm^2^ were used to produce hard TAs and those requiring compression forces ranging between 3.5 and 2.1 mN/mm^2^ were used to produce soft TAs.^10^ After consistency measurements, mixtures were used to produce TAs of 2.5 and 3 mm in diameter and each TA type was produced in lengths of 5 and 10 mm. TAs of 2.5 mm were used to mimic M2 and M1-M2 occlusions and TAs of 3 mm were used to mimic M1 occlusions.

### In vitro DTA experiments

Two operators with experience in mechanical thrombectomy (MTB) (GB and PM) performed in vitro DTA experiments with TAs located in M1 and M1-M2 (in such cases, TAs were mainly located in M2, but their proximal edges protruded in the distal portion of M1) and M2 segments (TAs were located in the distal portion of M2) (figure 1B–D). Five DTA procedures were performed for each TA location, consistency, and length; each procedure consisted of up to three DTA attempts. In cases of DTA failure (presence of any residual TA or distal emboli), a STR thrombectomy was performed as a rescue technique.

A large bore AC (SOFIA 6F Plus, MicroVention, Aliso Viejo, CA, USA) was used to perform DTA experiments for M1 and M1-M2 occlusions, while a small bore AC (3MAX Penumbra Inc., Alameda, CA, USA) was used for M2 occlusions. Large bore ACs were introduced into the phantom via a standard 8F guide catheter (AXS Infinity LS; Stryker, Kalamazoo, MI, USA) and advanced up to the proximal edge of the TA. The AC formed with the TA an AOI of 180° for M1 occlusions and of 70°, 125.5°, or 160° for M1-M2 occlusions, according to the M2 segment in which the TA was located. For M2 occlusions, the small bore AC was navigated within the occluded M2 branch via a large bore AC maintained in the M1 segment and placed in contact with the proximal edge of the TA. In all M2 occlusion cases, the small bore AC and the TA were aligned (180°). During DTA, anterograde flow through the vascular phantom was not arrested so as not to interfere positively with DTA efficacy.

DTA was performed using an automated vacuum system (Stryker Medela AXS; Stryker) according to the following procedure: once placed in contact with the proximal edge of the TA, the ACs were connected to the vacuum system by a tube clamped for 90 s after the activation of the pump in order to obtain a suction force of around −87 kPa. The connecting tube was then unclamped to suddenly transmit negative pressure through the ACs. In the case of effective DTA, the TA was either aspirated through the AC into the vacuum system canister or remained corked at the distal end of the AC. When involving M1/M1-M2 occlusions in the latter case, the large bore AC was retrieved through the guide catheter out of the vascular phantom, while concomitant manual aspiration was applied through the guide catheter using a 60 mL syringe (VacLock, Merit Medical Systems, South Jordan, UT, USA). In cases of M2 occlusions, the small bore AC connected to the vacuum system and the corked TA were retrieved into the large bore AC placed in the proximal portion of M1, while manual aspiration was applied through it.

The DTA procedure was considered as a failure after three unsuccessful attempts and a STR-based MTB was then performed as a rescue technique. In such cases, regardless of the initial TA location, the AC was maintained in the proximal M1 portion and a STR (Solitaire FR 4–20 mm; Medtronic, Irvine, CA, USA) was deployed over the TA via a 0.021 inches microcatheter (Headway; MicroVention). Subsequently, the stent was completely retrieved inside the AC, which was maintained in the proximal portion of the M1 in order to allow a coaxial entry of the STR into the AC, while concomitant aspiration performed by the vacuum system was applied through it.

### Statistical analysis

The influence of three independent variables, that is, the AOI between the AC and the TA (70°, 125.5°, or 160°), consistency of the TA (soft or hard), and length of the TA (short or long) was evaluated by ordinal logistic regression with regard to the number of attempts needed to aspirate the TA located in M1, M1-M2, and M2 segments of the vascular phantom. In cases where the model fitting information retrieved a non-significant P value, where the goodness-of-fit tests were unfavorable (significant P values), or when unexpected singularities in the Fisher information matrix were found due to an independent variable, the results obtained by ordinal logistic regression were not upheld, and Fisher’s exact test was performed instead to test for independence between the variable concerned and the outcome variable. The significance level was defined as 0.05 (two-tailed) for all tests. All analyses were performed on SPSS V.22.

## Results

Results of in vitro DTA experiments are reported in table 1.

**Table 1:** Results of thromboaspiration procedures*

Angle of interaction	Soft short TA % (n)	Soft long TA % (n)	Hard short TA % (n)	Hard long TA % (n)
DTA success for M1 occlusions				
180°	100% (1)	100% (1)	100% (1)	100% (1)
DTA success for M1-M2 occlusions				
70°	100% (1)	100% (1)	80% (1.7)	0%
125.5°	100% (1)	100% (1)	100% (1)	80% (2.8)
160°	100% (1)	100% (1)	100% (1)	100% (1.7)
DTA success for M2 occlusions				
70°	0%	60% (2)	0%	0%
125.5°	80% (1.4)	100% (1)	100% (1.2)	60% (2.2)
160°	100% (1)	100% (1)	100% (1)	100% (1)

*In parentheses are the average number of passes performed to obtain complete thrombus analog removal.

DTA, direct thromboaspiration; TA, thrombus analog.

### M1 occlusions

Overall, DTA was effective at first pass in cases of M1 occlusion (20/20), regardless of TA consistencies and lengths.

#### Soft TAs

Soft TAs were more prone to be completely ingested into the AC when the tube connecting the AC and the vacuum system was unconstrained (P<0.001). Eight soft TAs (8/10) were completely ingested, while two remained corked at the distal tip of the AC.

#### Hard TAs

By contrast, hard TAs remained corked at the distal tip of the AC in all cases and were retrieved with the AC outside the phantom. TA length was not associated with aspiration success for M1 occlusions (P=0.650).

### M1-M2 occlusions

#### Soft TAs

DTA was effective overall for soft short and long TAs in M1-M2 cases, regardless of the AOI value (30/30).

#### Hard TAs

Moreover, DTA was effective for hard short TAs with AOI values of 125.5° and 160°, while 1/5 (20%) procedures failed with an AOI of 70°. DTA was always ineffective (5/5) for hard long TAs located in M1-M2 with an AOI of 70°. However, it was effective in 4/5 (80%) procedures with an AOI of 125.5° and always effective with an AOI of 160° (5/5).

Overall, in experiments where the TA was located in M1-M2, the AOI was found to significantly affect the need for additional passes to successfully aspirate the TA (P=0.013), with larger angles associated with fewer attempts required (odds ratio (OR) 0.976; 95% confidence interval (CI) 0.958 to 0.995). Accordingly, with all other factors being equal, for a reduction in 1° in the AOI between the catheter and the TA, the probability of one additional attempt needed to aspirate the TA was 0.024 or 2.4%. Therefore, an AOI of 125.5° was 53 times less likely to require an additional aspiration attempt than an AOI of 70°, and an AOI of 160° was 34 times less likely to require additional attempts than a 125.5° AOI. TA length also significantly affected the need for further attempts at TA aspiration (P=0.003) with an OR of 13.93 (95% CI 2.52 to 76.89).

Similarly, TA consistency also had an influence on the number of attempts needed to aspirate the TA. Hard TAs were hence almost 14 times more likely to require further passes with 28/30 (93.3%) of soft TAs versus 18/30 (60%) of hard TAs being aspirated after one single attempt. The association between TA consistency and attempts needed to aspirate the TA was investigated by Fisher’s exact test. A highly significant result (P<0.001) was found with soft TAs strongly associated with fewer attempts at TA aspiration. All (30/30) (100%) soft TAs as opposed to only 16/30 (53.3%) hard TAs were successfully aspirated after the first attempt. Regarding hard TAs, 3/30 (10%) needed two aspiration attempts, 3/30 (10%) three attempts, and 8/30 (26.7%) could not be successfully aspirated even after three attempts.

### M2 occlusions

For M2 occlusion DTA experiments, the small bore ACs were placed in contact with the proximal edge of the TA located in one of the M2 segments. In all cases, the AC and the TA formed an AOI of 180°, regardless of the angle formed by the respective M2 branch with M1. Similar to DTA experiments performed with large bore ACs, once the highest negative pressure value was reached by the vacuum system, the tube connected to the AC was unconstrained and the TA was either aspirated through it or remained corked at its distal tip.

#### Soft TAs

Only 3/10 soft TAs were fully aspirated by the 3 MAX.

#### Hard TAs

All other hard and soft TAs were corked on the 3 MAX tip. In such instances, the AC and the corked TA were retrieved from M2 to M1 in order to be received into a large bore AC located in M1.

Along the retrieval process, the AOI between the small bore ACs and TAs varied from 180° to 70°, 125.5°, or 160°, according to the M2 branch in which the TA was originally located, and such a variation affected the probability of success of the procedure (P<0.001) with an OR of 1.084 (95% CI 1.041 to 1.129). As a consequence, increasing the AOI by 1° increased the probability of aspiration success by 0.084 or 8.4%.

Overall, in 70° M2 branch occlusion experiments (10/10), DTA failed in removing hard short and long TAs and soft short TAs (5/5), while it was effective for soft long TAs in 3/5 (60%) experiments. In 8/10 (80%) procedures, DTA was effective in 125.5° M2 branch occlusion experiments for hard TAs (5/5, short; 3/5, long), while soft TAs were aspirated in 9/10 experiments (4/5, short; 5/5, long; 90%). DTA was effective overall in 160° M2 occlusion experiments (20/20), irrespective of TA consistency and length.

### Rescue STR technique

In cases of DTA failure, a STR-based MTB combined with DTA was performed as a rescue technique. Overall, the STR rescue technique was effective in 25/27 (92.6%) cases.

For M1-M2 occlusions, a STR rescue technique was needed in 7/60 (11.6%) cases. Among these seven cases, STR rescue was needed in five cases for an M1-70° M2 occlusion due to a hard long TA and effective in four cases. The remaining two cases were related to a short hard TA for M1-70° M2 and a long hard TA for M1-125.5° M2 and treatment was effective in both.

In the case of M2 occlusions, STR rescue treatment was needed in 20/60 (33.3%) tests as follows: for M1-70° M2: 5/5 soft short TAs; 2/5 soft long TAs; 5/5 hard short TAs; and 5/5 hard long TAs. For M1-125.5° M2: 1/5 soft short TAs and 2/5 hard long TAs. For M1-160° M2: 0/20. STR rescue treatment was effective in 19/20 (95%) cases and failed only in one (1/5) case in which a long hard TA occluded a 70° M2 branch.

## Discussion

This in vitro study confirmed that DTA efficacy was related to the AOI formed by the AC and the TA. Moreover, STR thrombectomy was confirmed to be a valid rescue technique, even in the presence of an unfavorable AOI. Together with the evaluation of different AOIs, our experiments allowed us to evaluate how additional variables, such as TA consistency and size, influenced DTA efficacy.

A number of clinical and in vitro studies analyzed factors influencing the effectiveness of DTA. Boisseau *et al* 11 assessed the impact of the diameter of the aspiration catheter and the interaction between the clot and the aspiration catheter (AOI) on DTA, as well as the composition and consistency of the clot. Furthermore, additional reports evaluated the influence of aspiration variations, such as cyclical aspiration to DTA.^12^

We observed that AOIs formed between the large bore AC and the TA of around 180° encountered in M1 occlusions (AOI=180°) and M1-160° M2 occlusions (AOI=160°) were related to high DTA efficacy. We hypothesized that the vector of the suction force (inside the AC) was aligned to the main axis of the TA in such configurations and, consequently, the suction force was entirely transferred along the TA. Nevertheless, even in the presence of such favorable AOIs, DTA efficacy and the number of passes needed to be effective were as also related to TA consistency and size. Indeed, short soft TAs were more prone to fragment during aspiration and were completely ingested inside the AC, while long hard TAs tended to remain corked at the distal tip of the AC. In such instances, the AC was retrieved outside the vascular phantom. Thus, TA removal success was finally related to the balance between the aspiration force that maintained the TA fastened at the distal tip of the catheter and opposite forces (friction) encountered along the retrieval, which acted to displace the TA from the distal tip. Experiments showed that DTA efficacy decreased for acute AOIs, such as those encountered in M1-70° M2 occlusions (AOI=70°). In such a configuration, the vector of the suction force was misaligned to the main axis of the TA and consequently the suction force was transmitted only to the proximal portion of the TA and not along its entire axis. Nevertheless, even in this configuration, TA consistency and size influenced DTA efficacy with hard TAs never removed and soft TAs sporadically removed.

In experiments involving M2 occlusions, some unusual DTA results were observed. In such a configuration, the AOI between the small bore AC and the TA was around 180° at the beginning of the procedure and the catheter was able to either aspirate the TA (especially soft TAs) or maintain the TA corked at the distal tip of the AC. In the latter instance, as described for the large bore AC, the small bore AC was extracted from the phantom and the TA was often lost during retrieval when the AOI between the AC and the TA changed over the angle formed by the concerned M2 branch and M1. Such behavior was mainly observed for 70° M2 branch occlusions where the AC catheter was retrieved over an acute angle (70°), with all hard TAs (10/10) corked at the distal tip of the AC after the activation of the vacuum system and subsequently lost during retrieval over the M1 −70° M2 convergence.

In cases of DTA failure, the STR rescue technique was highly effective in our experiments. In such instances, the large bore AC was maintained in the proximal portion of the M1 segment of the phantom and used to deliver a STR over the TA; subsequently, the STR was able to be completely retrieved inside the AC during concomitant aspiration. The rationale of this technique, based on our clinical practice, was to use the STR to draw the TA close to the distal tip of the AC in order to reproduce a more favorable AOI with the AC, thus allowing a better transmission of the suction force to the TA. The results of our in vitro study are in agreement with those already observed by our clinical team and could explain those reported in several clinical studies aimed at evaluating the performance of DTA.^9 11 13–15^ These studies have demonstrated high DTA efficacy for M1 and basilar artery occlusions. Indeed, M1 and the basilar artery frequently have a straight geometry, determining a coaxial interaction between the AC and the clot and thus a favorable AOI. Furthermore, previous clinical reports have already reported the superiority of STR-based thrombectomy compared to DTA performed with low-profile ACs in cases of M2 occlusions.^16–18^ Finally, our results confirmed the high rate of first-pass recanalization obtained with combined STR and DTA (rescue procedure in our experiments) as previously reported.^19–21^

## Conclusions

This in vitro study confirmed that the AOI formed by the AC and the TA influenced DTA efficacy. An AOI >125.5° was related to an effective DTA, even in the presence of a large hard TA. Conversely, an unfavorable AOI could be related to DTA failure, even in the presence of a small soft TA. Finally, STR thrombectomy was proven to be an effective rescue treatment after DTA failure, even in the presence of an unfavorable AOI.

## Data Availability

All data are available upon request to the corresponding author.
